# Description of *Ellipsomyxa prima* n. sp. in the gallbladder of *Gambusia yucatana* (Cyprinodontiformes: Poeciliidae) from freshwater springs in the Yucatán Peninsula, Mexico

**DOI:** 10.1038/s41598-025-10781-w

**Published:** 2025-07-12

**Authors:** Graciela Colunga-Ramírez, Gábor Cech, M. Leopoldina Aguirre-Macedo, Kálmán Molnár, Csaba Székely, Boglárka Sellyei

**Affiliations:** 1 HUN-REN Veterinary Medical Research Institute, Budapest, Hungary; 2 Doctoral School of Animal Biotechnology and Animal Science, Hungarian University of Agriculture and Life Sciences, Gödöllő, Hungary; 3 Centro de Investigación y de Estudios Avanzados del Instituto Politécnico Nacional, Departamento Recursos del Mar, Unidad Mérida, Carretera Antigua a Progreso Km 6, Cordemex, Mérida, 97310 Yucatán Mexico

**Keywords:** Cnidaria, Fish parasites, Myxozoa, Phylogeny, Poeciliid fish, Parasitology, Parasite biology, Parasite genetics

## Abstract

The Celestún Lagoon is located on the coast of the Yucatán Peninsula in southern Mexico. The peripheral margins of the lagoon are exposed to a constant influx of water from freshwater springs, where a wide diversity of freshwater fishes occurs. Simultaneously, these freshwater springs are mixing with marine water from the sea and brackish water from the lagoon. *Gambusia yucatana* is an endemic poeciliid freshwater fish of this coastal region, with no current parasitological records. In December 2023, 24 specimens from two freshwater springs near the Celestún Lagoon were examined for myxozoan infection. Disporic plasmodia containing mature myxospores floating freely within the bile were detected in all fish. The myxospores were ellipsoidal in the valvular and sutural views, measuring 9.5 ± 0.6 μm length and 6.5 ± 0.5 μm width. Two pyriform polar capsules discharging on opposite sides, 3.2 ± 0.4 μm length and 2.3 ± 0.2 μm width, and 5–6 polar filament coils. Polar tubule 31.3 μm long and coiled 5–6 times. The partial sequences of 18S rDNA and 28S rDNA displayed a similarity of ≤ 96.4% and ≤ 85.2% to all other available sequences from the genus *Ellipsomyxa*. Phylogenetic analyses revealed that the novel species named *Ellipsomyxa prima* n. sp. is closely related to other congeneric *Ellipsomyxa* parasites. This study describes the first parasite in *G*. *yucatana* and the first *Ellipsomyxa* species reported in a fish of the order Cyprinodontiformes and the family Poeciliidae.

**Supplementary Information:**

The online version contains supplementary material available at 10.1038/s41598-025-10781-w.

## Introduction

Myxozoans (Cnidaria) constitute a group of fish parasites, with more than 2,600 species classified into 64 genera described worldwide^1–3^. A total of 12 nominal myxozoan species belonging to 6 genera have been identified in Mexico^2,4^. The most recent description was provided by Colunga-Ramírez et al.^4^, who reported *Myxobolus mayarum* Colunga-Ramírez, Aguirre-Macedo, Molnár, Székely, Sellyei, Cech, 2025 and *Kudoa mayarum* Colunga-Ramírez, Aguirre-Macedo, Molnár, Székely, Sellyei, Cech, 2025, parasitizing the cichlid fish *Mayaheros urophthalmus* (Günther, 1862), an endemic freshwater species of the Yucatán Peninsula, Mexico. *Ellipsomyxa papantla* Alama-Bermejo, Hernández-Orts, García-Varela, Oceguera-Figueroa, Pecková & Fiala, 2023, infecting *Dormitator maculatus* (Bloch, 1792), is the sole member of the genus *Ellipsomyxa*, Køie, 2003, thus far described in Mexico^2^. The genus *Ellipsomyxa* is a member of the class Myxozoa (Køie 2003), comprising only 23 species that infect the gallbladder of marine and freshwater fishes globally^5,6^. However, no species of *Ellipsomyxa* have been reported in fish belonging to the order Cyprinodontiformes Berg, 1940, including those in the family Poeciliidae Bonaparte, 1831. The family Poeciliidae includes over 40 species of the genus *Gambusia* Poey, 1854, which are found in freshwater habitats and often occur in salt and brackish water bodies, estuaries, coastal lagoons, or along shores in mangrove areas^7,8^. *Gambusia yucatana*, Regan, 1914, is an endemic fish species from the Yucatán Peninsula, Mexico^9^, where the Celestún Biosphere Reserve is located, and includes the brackish Celestún Coastal Lagoon, known for its extensive ichthyofauna^10,11^. *Gambusia yucatana* is of ecotoxicological importance due to its sensibility to petroleum components such as polycyclic aromatic hydrocarbons (PAHs) and pesticides; as a result, it has been proposed as a sentinel organism^12,13^. Despite its importance, no parasites infecting *G*. *yucatana* have been described. In the present study, a myxosporean infection was observed in the gallbladder of *G*. *yucatana* collected in the Baldiosera and Ya´xaa freshwater springs adjacent to the Celestún Coastal Lagoon in the Yucatán Peninsula, Mexico. Based on morphological characteristics and molecular analyses of 18S and 28S rDNA sequence data, we describe the first *Ellipsomyxa* species to infect a poecilid fish and the first parasite reported in *G*. *yucatana*.

## Materials and methods

### Ethical statement

The experimental protocols together with fish handling and sampling were in accordance with the Guidelines for Care and Manipulation of Laboratory Animals of Cinvestav, the Mexican Official norm NOM-062-ZOO-199, and were also approved by the Institutional Animal Care and Use Committee of the Veterinary Medical Research Institute, Budapest, Hungary. All research involving experiments on fish (*Gambusia yucatana*) was reviewed and approved by the Hungarian National Scientific Ethical Committee on Animal Experimentation under reference number: .PE/EA/00081 − 4/2023. The authors complied with the ARRIVE guidelines (https://arriveguidelines.org).

### Sample collection

In December 2023, 24 *Gambusia yucatana* specimens were collected from two freshwater springs at the margin of Celestún Coastal Lagoon, Baldiosera, and Ya´xaa (Table 1). These springs are located within the Celestún Biosphere Reserve in the Yucatán Peninsula, México, in proximity to the brackish water of the Celestún coastal lagoon, and approximately 4.0 km from the sea.

**Table 1:** Localities of sample collection of *Gambusia yucatana* specimens from the Celestún biosphere reserve in the Yucatán Peninsula, Mexico (ppt = parts per thousand).

Host	No. of fish	Water spring	Coordinates	Salinity (ppt)	Temperature (ºC)
*Gambusia yucatana*	17	Baldiosera	20° 87′ 74′ N, 90° 35′ 54′ W	Surface 2.1 Ground 5.1	Surface 26.5 Ground 26.5
	7	Ya´xaa	20° 87′ 74′ N, 90° 35′ 54′ W	Surface 15.2 Ground 26.3	Surface 24.5 Ground 23.5

The fish were caught using nets and were transported alive in aerated tanks to the Aquatic Pathology Laboratory at the Centro de Investigación y de Estudios Avanzados, Unidad Mérida (Cinvestav-Mérida), where they were kept in an aerated aquarium. The specimens were examined for myxosporean infections. The mean total length and mean fish body weight were 2.4 ± 0.36 cm (1.6–3.0 cm) and 0.14 ± 0.06 g (0.04–0.26 g). Due to their diminutive fish body size, they were euthanised by brain puncture to avoid mechanical damage to the integrity of the organs. The gills, gastrointestinal tract, gallbladder, muscle, and fins were studied under a stereomicroscope (Olympus SZX16) and excised pieces of fresh tissue were mounted on glass slides, then compressed under a cover glass and observed via light microscopy (Olympus BX53 with a digital camera Olympus DP74). Smear preparations of infected bile, either fresh or Lugol-stained, were used for morphological identification of the parasite. Additionally, samples from four parasitized gallbladders were preserved in molecular-grade 96% ethanol for subsequent molecular analyses.

### Morphological identification

Fresh and Lugol-stained bile smears of infected bile were observed under a light microscope. The morphological and morphometric data were determined according to Whipps & Font^14^ using ImageJ software (http://imagej.nih.gov/ij). All measurements are given in micrometers (µm) expressed as the mean and standard deviation, followed by the range in parentheses.

### Molecular characterization

Ethanol-fixed gallbladders with *Ellipsomyxa* myxospores were rinsed three times in a Tris-HCl buffer (10 mM Tris-HCI, pH 8.5), and then 50 µl nuclease-free water was added. Genomic DNA was isolated from the rinsed gallbladders using the Genomic DNA Mini Kit (Geneaid Biotech Ltd., Taiwan), following the instructions of the manufacturer. The small subunit ribosomal DNA (18S rDNA) and large subunit ribosomal DNA (28S rDNA) were amplified in short overlapping fragments by polymerase chain reaction (PCR) using different combinations of specific myxozoan primers (Table 2). The PCR reactions were performed in a final volume of 25 µl, which contained 1 × DreamTaq buffer (10 ×; Thermo Scientific), 0.2 mM dNTP mix (10 mM; Thermo Scientific), 100 nmol L-1 of each primer, 0.5 U DreamTaq polymerase (5 U; Thermo Scientific), 1 µl of template DNA, and nuclease-free water. The reaction conditions for the 18S rDNA were 95 °C for 5 min, followed by 35 cycles of 95 °C for 30 s, 55 °C for 1 min, and 72 °C for 90 s, with a final extension at 72 °C for 10 min. The reaction conditions for the 28S rDNA were 94ºC for 3 min, followed by 35 cycles of 94ºC for 45 s, 64ºC for 1 min, 72ºC for 1 min 45 s, with a final extension at 72ºC for 7 min. The amplified PCR products were run on a 1% agarose gel with 1× Tris-acetate-EDTA (TAE) buffer, stained with ethidium bromide (0.5 µl/ml) and examined under a UV transilluminator. The appropriate-sized bands (Supplementary Fig. 1) were purified with the DNA Fragment Purification Kit (Invitek, Berlin, Germany). Each PCR product was Sanger sequenced in both forward and reverse directions using a BigDye Terminator v3.1 Cycle Sequencing Kit (Applied Biosystems, Foster City, USA) and run on an ABI PRISM 3100 Genetic Analyser (Applied Biosystems).

**Table 2:** Primers used for amplifying and sequencing the 18S rDNA and 28S rDNA genes of *Ellipsomyxa prima* n. sp. F: forward, and R: reverse.

Gene	Primer	Application	Sequence (5′–3′)	References
18S rDNA	F: Myx1F^1^	PCR and sequencing	GTGAGACTGCGGACGGCTCAG	^15^
	F: Myxgen4F^2^	PCR	GTGCCTTGAATAAATCAGAG	^16^
	F: ACT1F^3^	PCR and sequencing	TTGGGTAATTTGCGCGCCTGCTGCC	^15^
	R: ACT1Fr	Sequencing	TTGGGTAATTTGCGCGCCTGCTGCC	^15^
	R: ERB10^2,3^	PCR and sequencing	CTTCCGCAGGTTCACCTACGG	^17^
	R: MyxospecR^1^	PCR	GGTTTCNCDGRGGGMCCAAC	^18^
	R: SphR	Sequencing	GTTACCATTGTAGCGCGCGT	^19^
28S rDNA	F: Kt28S1F^4^	PCR and sequencing	CAAGACTACCTGCTGAAC	^20^
	F: Myxo28S1F^5^	PCR and sequencing	AGTAACTGCGAGTGAAGYG	^20^
	F: NLF1050^6^	PCR and sequencing	AATCGAACCATCTAGTAGCTGG	^21^
	F: NLF1260^7^	PCR and sequencing	ACCTCCACTCAGGCAAGATTA	^21^
	F: NLF184^8^	PCR and sequencing	ACCCGCTGAAYTTAAGCATAT	^22^
	R: NLR1270^8^	PCR and sequencing	TTCATCCCGCATCGCCAGTTC	^21^
	R: NLR3113^6^	PCR and Sequencing	GTCTAAACCCAGCTCACGTTCCCT	^22^
	R: 28S3R^4,5,7^	PCR and sequencing	GAGCACTGGGCAGAAATC	^23^

The primer pair combinations employed for PCR are indicated by numbers in the superscripts.

### Phylogenetic analyses

The sequences were checked and edited according to chromatograms in Chromas v. 2.6.6 software (Technelysium Pty Ltd., Queensland, Australia), assembled, and aligned in MEGA11 software^24^. To explore the phylogenetic relationships of the *Ellipsomyxa* in study to other myxozoans, all congeneric sequences available in the GenBank database were downloaded, plus 17 sequences from closely related myxosporean species. In the case of the 28S rDNA, three congeneric sequences available in the GenBank database were added to the analysis, along with 13 additional sequences from closely related myxosporean species. The 18S rDNA *Chloromyxum leydigi* Mingazzini, 1890 (ON383909) and the 28S rDNA sequence of *C*. *leydigi* (FJ417055) were used as outgroups. The 18S rDNA sequences were aligned with MAFFT v. 11 online servers^25^ using the G-INS-i strategy selected. In turn, 28S rDNA sequences were aligned using ClustalW^26^ in MEGA11. Poorly aligned positions were eliminated using Gblocks v. 0.91b with less stringent parameters^27^.

Phylogenetic trees of the 18S rDNA and 28S rDNA sequences were constructed using Maximum Likelihood (ML) and Bayesian inference (BI) under the general time reversible model with a gamma-distributed rate and invariant sites (GTR + G + I) based on the Akaike information criterion (AIC) in MEGA11^24^. Maximum likelihood analyses were performed using MEGA11, and bootstrap (BS) values were calculated from 1000 replicates. Bayesian Inference analyses were performed in MrBayes v. 3.2^28^, and posterior probabilities (PP) were calculated over 10 million generations with four parallel chains running simultaneously every 1000 generations with the burn-in set at 25%. The ML and BI trees were displayed and annotated in v. MEGA11 and FigTree v. 1.4.4^29^, respectively. The two phylogenetic trees were edited with CorelDRAW Graphics Suite 2019 v. 21.3.0.755 (Corel Corporation, Ottawa, Canada). Genetic distances between the species in this study and other *Ellipsomyxa* sequences available in GenBank, all 19 ones used above, were calculated with the *p*-distance model in MEGA11^24^.

## Results

All (*n* = 24) examined *G. yucatana* specimens exhibited disporic plasmodia and mature myxospores floating freely within the bile, with morphological characteristics consistent with those of the genus *Ellipsomyxa*. The morphological and sequence analyses showed that the myxospores described here belong to a novel *Ellipsomyxa* species. The species description is provided below.

### Description

#### *Ellipsomyxa prima* n. sp.

**Fig. 1:**
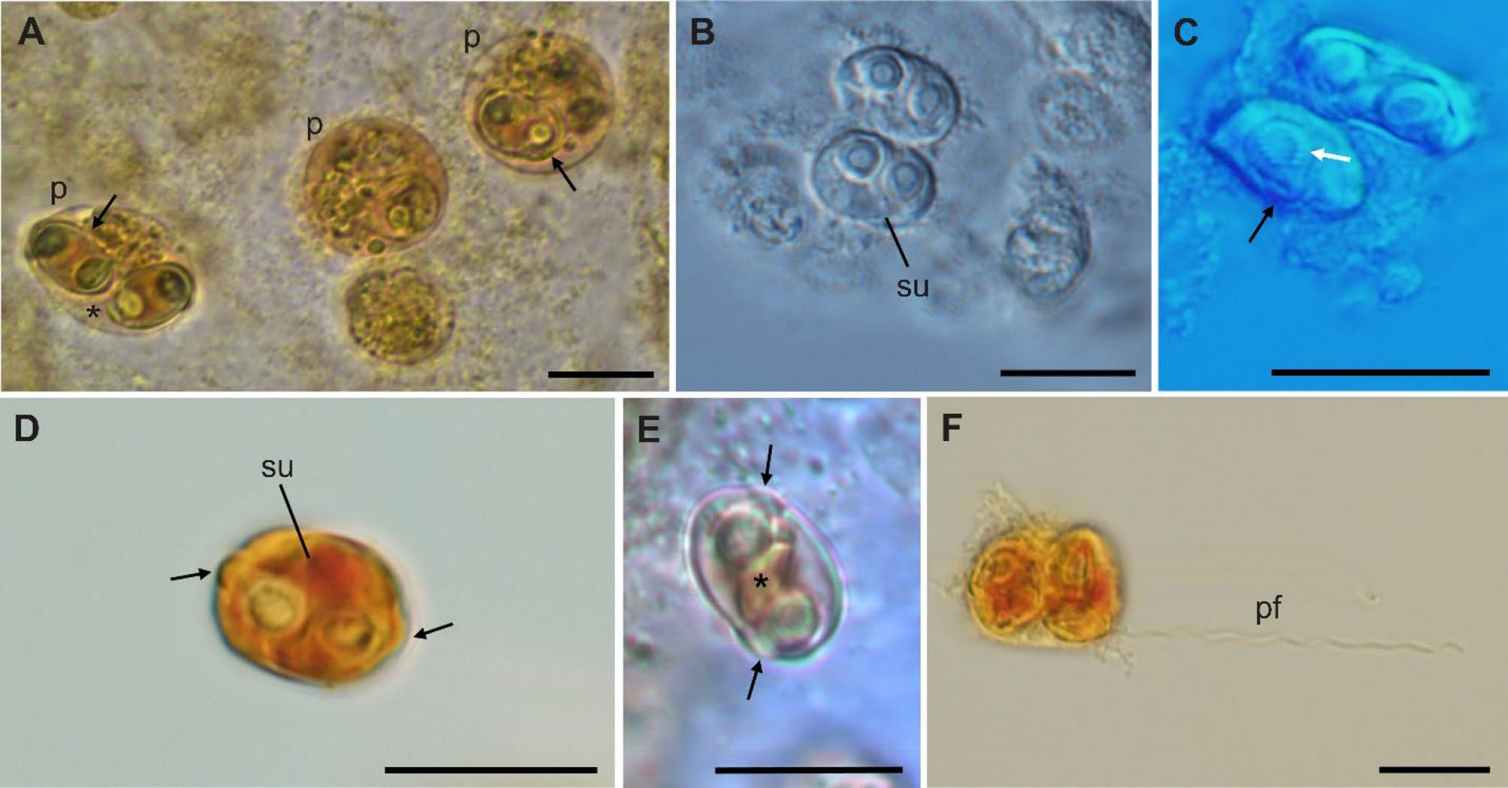
*Ellipsomyxa prima* n. sp. plasmodia and mature myxospores in the gallbladder of *Gambusia yucatana*. (**A**) Spherical disporic plasmodia (p) containing myxospores (arrows) and immature plasmodia (ip); Ellipsoid plasmodia (*). (**B**) Myxospores in sutural view showing a sutural line (su). (**C**) Myxospore showing the polar tubule of a polar capsule coiling 5 − 6 times (white arrow). (**D**) Lugol stained myxospores in sutural view evidencing the capsular foramina (arrows). (**E**) Myxospore in sutural view highlighting the sporoplasm (*). (**F**) Lugol stained myxospore showing one polar tubule extruded (pt). Scale bars = 10 μm.

**Fig. 2:**
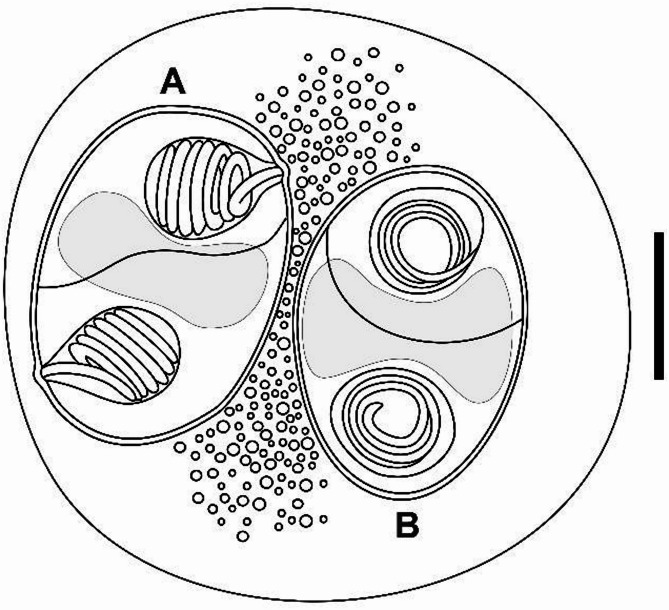
Line drawing of disporic plasmodia with two mature myxospores of *Ellipsomyxa prima* n. sp. (**A**) Sutural view (**B**) frontal view. Scale bar = 10 μm.

Plasmodia (*n* = 14): Disporic, subspherical to spherical in shape, measuring 15.5 ± 2.2 (13.0–20.5) µm in length and 14 ± 1.9 (11.8–19.0) µm in width (Figs. 1 and 2). Ellipsoidal plasmodia rarely observed (Fig. 1A).

Spores (*n* = 42): Myxospores are ellipsoid in frontal and sutural views, measuring 9.51 ± 0.6 (8.3–10.9) µm in length, 6.5 ± 0.5 (5.2–7.6) µm in width, and 4.1 ± 0.5 (3.60–4.9) µm in thickness, with a curved suture line (Fig. 1; Table 3). Two pyriform and equal-sized polar capsules discharging sub-laterally to opposite sides in the sutural view, measuring 3.2 ± 0.4 (2.5–4.5) µm length and 2.3 ± 0.2 (1.7–3.0) µm width. Sporoplasm situated between the polar capsules (Fig. 1E). Polar tubule coiling 5–6 times (Fig. 1C), extruded polar filament (*n* = 1) 31.39 μm long (Fig. 1F).

**Table 3:** Morphological comparison of myxospores dimensions of *Ellipsomyxa prima* n. sp. and related species.

Species	Fish host	Length	Width	Thickness	PCM	PCL	PCW	PTC	PTL	References
*Ellipsomyxa prima* n. sp.	***Gambusia yucatana***	**9.5 ± 0.6 (8.3–10.9)**	**6. 5 ± 0.5 (5.2–7.6)**	**4.1 ± 0.5 (3.6–4.9)**	**P**	**3.2 ± 0.4 (2.5–4.5)**	**2.3 ± 0.2 (1.7–3.0)**	**5–6**	**31.3**	**Present study**
*Ellipsomyxa ariusi*	*Arius arius*	10.1 ± 0.8	6.8 ± 0.5	7.7 ± 0.7	P	2.8 ± 0.3	2.5 ± 0.4	4–5	32.2 ± 2.1	^30^
*Ellipsomyxa apogoni*	*Apogon doederleini*	10.1 ± 0.6 (9.1–12.0)	6.9 ± 0.3 (6.4–7.5)	*–*	P	3.7 ± 0.5 (2.9–4.8)	2.7 ± 0.3 (2.1–3.4)	2–4	*–*	^31^
*Ellipsomyxa boleophthalmi*	*Boleophthalmus dussumieri*	9.8 ± 0.5 (9.0–10.7)	7.2 ± 0.6 (6.0–7.8)	*–*	S	2.8 ± 0.3 (2.6–2.8)	*–*	3–4	32.6 (20.5–40.8)	^32^
*Ellipsomyxa gordeyi*	*Mugil cephalus*, *Planiliza melinoptera*, *Planiliza sp.* D sensu, *Planiliza subviridis*, *Gobiosoma bosc*	9.5 ± 0.6 (8.7–10.9)	7.0 ± 0.5 (6.2–8.1)	*–*	P	3.5 ± 0.4 (2.7–4.3)	2.4 ± 0.2 (2.0–2.7)		39.2–40.1	[5]
*Ellipsomyxa tucujuensis*	*Satanoperca jurupari*	10.11 (8.56–10.5)	7.81 (5.96–9.56)	*–*	S	3.12 (2.3–3.9)	2.5 (2.2–2.9)	*–*	*–*	^33^
*Ellipsomyxa adlardi*	*Gobiosoma bosc*	12.4 ± 0.2 (11.3–14.4)	7.7 ± 0.1 (7.1–8.8)	7.8 ± 0.2 (7.1–9.0)	P	4.3 ± 0.06 (3.9–4.9)	3.6 ± 0.03 (3.3–4.1)	5–6	–	^14^
*Ellipsomyxa amazonensis*	*Brachyplatystoma rousseauxii*	12.8 (12.3–13.6)	7.6 (6.7–8.7)	–	P	3.8 (3.8–4)	3.1 (2.5–3.4)	2–3	–	^34^
*Ellipsomyxa arariensis*	*Pygocentrus nattereri*, *Pimelodus ornatus*	12.6 (12–13.4)	7.3 (6.7–8)	–	P	3.5 (3.4–4)	2.6 (2.5–3.2)	5–6	–	^35^
*Ellipsomyxa arothroni*	*Arothron hispidus*	15.4 ± 1.3 (12.3–17.7)	11.7 ± 0.9 (9.9–13.6)	–	P	6.6 ± 0.6 (5.4–7.9)	4.5 ± 0.4 (3.9–5.4)	4–6	–	^31^
*Ellipsomyxa fusiformis*	*Sphyrna zygaena*	9	16	–	S	4.5	4.5	–	–	^36^
Ellipsomyxa gobii*	*Pomatoschistus microps*	7.0 (6.6–7.5)	8.7 (8.0–9.0)	11.6 (10.8–12.0)	S	3.1 (3.0–3.2)	3.1 (3.0–3.2)	6–7	40	^37^
*Ellipsomyxa gobioides*	*Gobioides broussonnetii*	6.8 ± 0.2 (6.5–7.0)	7.2 ± 0.4 (6.9–7.5)	13.1 ± 0.3 (12.8–13.5)	P	4.6 ± 0.3 (4.3–4.8)	2.5 0.3 (2.1–2.7)	5–6	32	^38^
*Ellipsomyxa intravesica*	*Pangasius macromena*	13.3 ± 0.9 (12.0–15.0)	8.4 ± 0.5 (8.0–9.0)	8.5 ± 0.5 (8.9–9.0)	P	3.7 ± 0.5 (3.0–4.0)	3.6 ± 0.5 (3.0–4.0)	5–7	–	^39^
*Ellipsomyxa kalthoumi*	*Chelon salien*	17.2 (13–21)	13.2 (10–15)	–	S	5.5 (5–6)	5.5 (5–6)	9	70	^40^
*Ellipsomyxa manilensis*	*Arothron manilensis*	15.2 ± 1.1 (13.8–17.1)	11.8 ± 1.1 (10.2–13.3)	–	S, P	5.6 ± 0.6 (4.6–6.6)	4.5 ± 0.3 (4.2–5.0)	3–4	–	^31^
*Ellipsomyxa matosi*	*Ageneiosus ucayalensis*	13.1 ± 1.0 (11.9–14.4)	8.0 ± 0.8 (7.2–9.4)	–	P	4.9 ± 0.3 (4.4–5.4)	3.2 ± 0.6 (26–3.9)	6–7	–	^6^
Ellipsomyxa mugilis*	*Chelon saliens*, *Chelon ramada*, *Mugil cephalus*	7.3 ± 0.8 (5.5–9.0)	6.8 ± 0.5 (5.5–8)	11.5 ± 0.9 (10–13.5)	S	2.9 ± 0.5 (2.7–4.0)	2.9 ± 0.5 (2.7–4.0)	5	–	^41^
*Ellipsomyxa nigropunctatis*	*Arothron nigropunctatus*	13.8 ± 1.1 (11.9–16.3)	9.9 ± 1.5 (8.0–12.9)	–	P	4.7 ± 0.6 (3.5–5.7)	3.6 ± 0.5 (2.8–4.6)	5	–	^31^
*Ellipsomyxa papantla*	*Dormitator maculatus*	12.9 ± 0.8 (11.6–15.0)	9.1 ± 0.5 (7.6–9.9)	7.3 ± 0.7 (6.1–8.2)	S, P	3.8 ± 0.5 (2.6–4.6)	3.3 ± 0.5 (2.2–4.2)	3–4	20.9–29.2	^2^
*Ellipsomyxa paraensis*	*Cichla monoculus*	11.5 (10.5–12.4)	7.5 (6.6–8.6)	–	P	3.2 (2.1–3.9)	2.6 (2–3.3)	2–3	–	^42^
*Ellipsomyxa plagioscioni*	*Plagioscion squamosissimus*	11.1 (10.2–12.8)	6.6 (5.6–7.6)	–	P	3.8 (3.2–4.4)	2.8 (2.3–3.3)	5–6	–	^42^
*Ellipsomyxa santarenesis*	*Satanoperca jurupari*	12.0 ± 3.2 (10.7–13.7)	7.6 ± 1.5 (5.6–8.2)	–	S	2.8 ± 0.4 (2.0–3.6)	2.8 ± 0.4 (2.0–3.6)	4–5	–	^43^
Ellipsomyxa syngnathi*	*Syngnathus typhle*,* S. rostellatus*	6.8 (6.3–7.2)	8.1 (7.2–8.6)	10.0 (9.0–10.8)	P	3.6 (3.2–4.1)	2.9 (2.7–3.2)	5–6	–	^44^

*PCM* polar capsule morphology (P = pyriform; S = spherical), *PCL* polar capsules length, *PCW* polar capsules width, *PTC* polar tubule coiled, *PTL* polar tubule length, dashes: no data.

Dimension are given in micrometres and expressed as the mean followed by the range in parentheses.

Following the designation of *E*. *prima* n. sp., the subsequent five species (bold and italics) are listed at the top based on their morphological and morphometric resemblance to the newly described species. The other species are listed in alphabetical order. Note that measurement inconsistencies are observed in some *Ellipsomyxa* species. Some authors used the longest myxospore measurement as the thickness rather than the length (*).

### Taxonomic summary

Type host: *Gambusia yucatana* Regan, 1914 (Cyprinodontiformes: Poeciliidae).

Site in host: Coelozoic, in the gallbladder.

Prevalence: 24/24 (100%).

Type locality: Baldiosera and Ya´xaa freshwater springs (20° 87′ 74′ N, 90° 35′ 54′ W) into the Celestún Coastal Lagoon (Biosphere Reserve) (20° 87′, 74′ N, 90° 35′, 54′ W).

Material deposited: Phototypes were deposited in the parasitological collection of the Zoological Department, Hungarian Natural History Museum, Budapest, Coll. No. HNMPCC-HNHM-PAR-72,085. The DNA samples are available in the Fish Pathology and Parasitology Lab for request. Contact information was added in the data availability section.

Representative sequences: 18S rDNA (1636 bp) and 28S rDNA (2216 bp) were submitted in GenBank (accession numbers: PV385047 and PV428871).

 Etymology: The name derives from the Latin “*prima”*, meaning “first”.

Remarks: The morphological and morphometric features of *E*. *prima* n. sp. and their congeners are compared in Table 3. All documented species of *Ellipsomyxa*, including the novel species in this study, have been reported to infect the gallbladder. Considering the total size and dimensions of *E*. *prima* n. sp. myxospore and its polar capsules, it was compared with the following species: *Ellipsomyxa ariusi* Chandran, Zacharia, Sathianandan & Sanil, 2020, *Ellipsomyxa apogoni* Heiniger & Adlard, 2014, *Ellipsomyxa boleophthalmi* Vandana, Poojary, Tripathi, Pavan-Kumar, Pratapa, Sanil & Rajendran, 2021, *Ellipsomyxa gordeyi* Yurakhno, Ha, & Whipps, 2024, and *Ellipsomyxa tucujuensis* Ferreira, da Silva, de Carvalho, Bittencourt, Hamoy, Matos & Videira, 2021. Nevertheless, the type host, locality, and some subtle morphological differences should be highlighted for differentiation.

The myxospores of *E*. *prima* n. sp. and *E*. *ariusi* overlapped in almost all measured dimensions, but the novel species was found to be significantly thinner (4.1 μm vs. 7.7 μm), and have more polar tubules coiled (5–6 vs. 4–5). Furthermore, *E*. *ariusi* was found infecting *Arius arius* (Hamilton, 1822), a marine fish from India. In comparison with *E*. *apogoni*, they have similar myxospore dimensions (9.5 × 6.5 μm vs. 10.1 × 6.9 μm), but differ in polar tubule coiling (2–4 vs. 5–6), which is less in *E*. *apogoni*. In addition, *E*. *apogoni* was collected from the marine fish *Apogon doederleini* (Jordan & Snyder, 1901) from Australia. Consistent with the *E*. *boleophthalmi*, both species occurred in fish inhabiting an estuarine environment, but *E*. *boleophthalmi* was found in *Boleophthalmus dussumieri* (Valenciennes) from India. The dimensions of both latter species are highly similar (9.5 × 6.5 μm vs. 9.8 × 7.2 μm); however, the morphology of the polar capsule is spherical for *E*. *boleophthalmi*, and pyriform in *E*. *prima* n. sp. It is similar to *E*. *tucujuensis*, which has polar capsules with a spherical shape.

The myxospores of *E*. *gordeyi* showed the greatest dimensional similarity to myxospores of *E*. *prima* n. sp. The absence of data on the thickness and polar tubule coiling prevents their morphological differentiation. The occurrence of *E*. *gordeyi* has been reported in different marine fish fish species from various families, including *Mugil cephalus* (Linnaeus), *Planiliza melinoptera* (Valenciennes), *Planiliza* sp. D sensu, *Planiliza subviridis* (Valenciennes), and *Gobiosoma bosc* (Lacepède) from Vietnam. Comparing the novel species with the unique *Ellipsomyxa* species described in Mexico; *E*. *papantla*, has been reported infecting *D*. *maculatus* in a freshwater environment. Morphologically, the myxospores of *E*. *prima* n. sp. are smaller in all dimensions.

However, measurements of *E*. *prima* n. sp. myxospores overlapped in almost all spore dimensions with the above mentioned species. The consensus 18S rDNA (1636 bp) and 28S rDNA (2216 bp) sequences of *E*. *prima* n. sp. obtained from *G*. *yucatana* did not reveal any significant match with myxozoan sequences available in GenBank. In the 18S and 28S rDNA phylogenetic analyses, *E*. *prima* n. sp. was not associated closely with any species. In the case of the 18S rDNA phylogeny, *E*. *prima* n. sp. is situated in a clade (PP = 0.89) that includes *Ellipsomyxa* species from freshwater, estuarine and marine habitats as well. The molecular data of this clade were employed to calculate the genetic *p*-distance and the sequence similarities. In the case of the 28S rDNA, sequences only from three *Ellipsomyxa* species were available (Table 4).

**Table 4:** Genetic *p*-distance (below the diagonal) and sequence similarities (in %, above the diagonal) of *Ellipsomyxa prima* n. sp. and closely related Sp.cies based on the 18S rDNA and 28S rDNA sequence data, followed by their respective GenBank accession numbers.

18S rDNA	(1)	(2)	(3)	(4)	(5)	(6)	(7)	(8)	(9)	(10)
(1) *Ellipsomyxa prima* n. sp. (PV385047)		96.8	96.4	96.4	96.3	96.2	96.2	95.8	95.6	95.5
(2) *Ellipsomyxa* sp. (MH212373)	0.032		97.1	97.1	96.7	97.0	97.4	97.3	96.3	97.4
(3) *Ellipsomyxa mugilis* (MK193812)	0.036	0.029		99.7	96.7	99.7	97.6	97.6	96.6	97.0
(4) *Ellipsomyxa syngnathi* (GQ229233)	0.036	0.029	0.003		96.5	99.6	97.5	97.6	96.5	96.9
(5) *Ellipsomyxa plagioscioni* (MT039013)	0.037	0.032	0.033	0.034		99.6	96.9	96.8	96.4	97.0
(6) *Ellipsomyxa gobii* (AY505127)	0.038	0.029	0.003	0.004	0.033		97.6	97.6	96.6	96.9
(7) *Ellipsomyxa adlardi* (JX443488)	0.038	0.026	0.024	0.025	0.031	0.024		99.2	97.2	96.8
(8) *Ellipsomyxa gordeyi* (PP296411)	0.041	0.027	0.023	0.024	0.031	0.023	0.007		97.5	96.8
(9) *Ellipsomyxa boleophthalmi* (MT611058)	0.043	0.036	0.033	0.034	0.036	0.033	0.027	0.024		96.9
(10) *Ellipsomyxa intravesica* (OR457656)	0.045	0.026	0.029	0.030	0.030	0.031	0.032	0.032	0.031	

28S rDNA	(1)		(2)		(3)		(4)			
(1) *Ellipsomyxa prima* n. sp. (PV428871)			85.2		84.4		82.8			
(2) *Ellipsomyxa adlardi* (PP309972)	0.1476				93.9		90.9			
(3) *Ellipsomyxa gordeyi* (PP309971)	0.1554		0.0607				92.3			
(4) *Ellipsomyxa gobii* (PP309973)	0.1714		0.0901		0.0762					

According to the 18S rDNA sequences, the highest sequence similarity was 96.4% (3.6% of genetic distance) with the of *Ellipsomyxa* sp. MH212373 (Unpublished) from the fish *Toxotes jaculatrix* (Pallas) in Terengganu, Malaysia, and 85.2% similarity (14.8% genetic distance) with the 28S rDNA sequence of *Ellipsomyxa adlardi* Whipps & Font, 2013 from the *Gobiosoma bosc* (Lacepède) in the estuarine Lake Pontchartrain, Louisiana (USA) (Table 4).

### Phylogenetic analyses

**Fig. 3:**
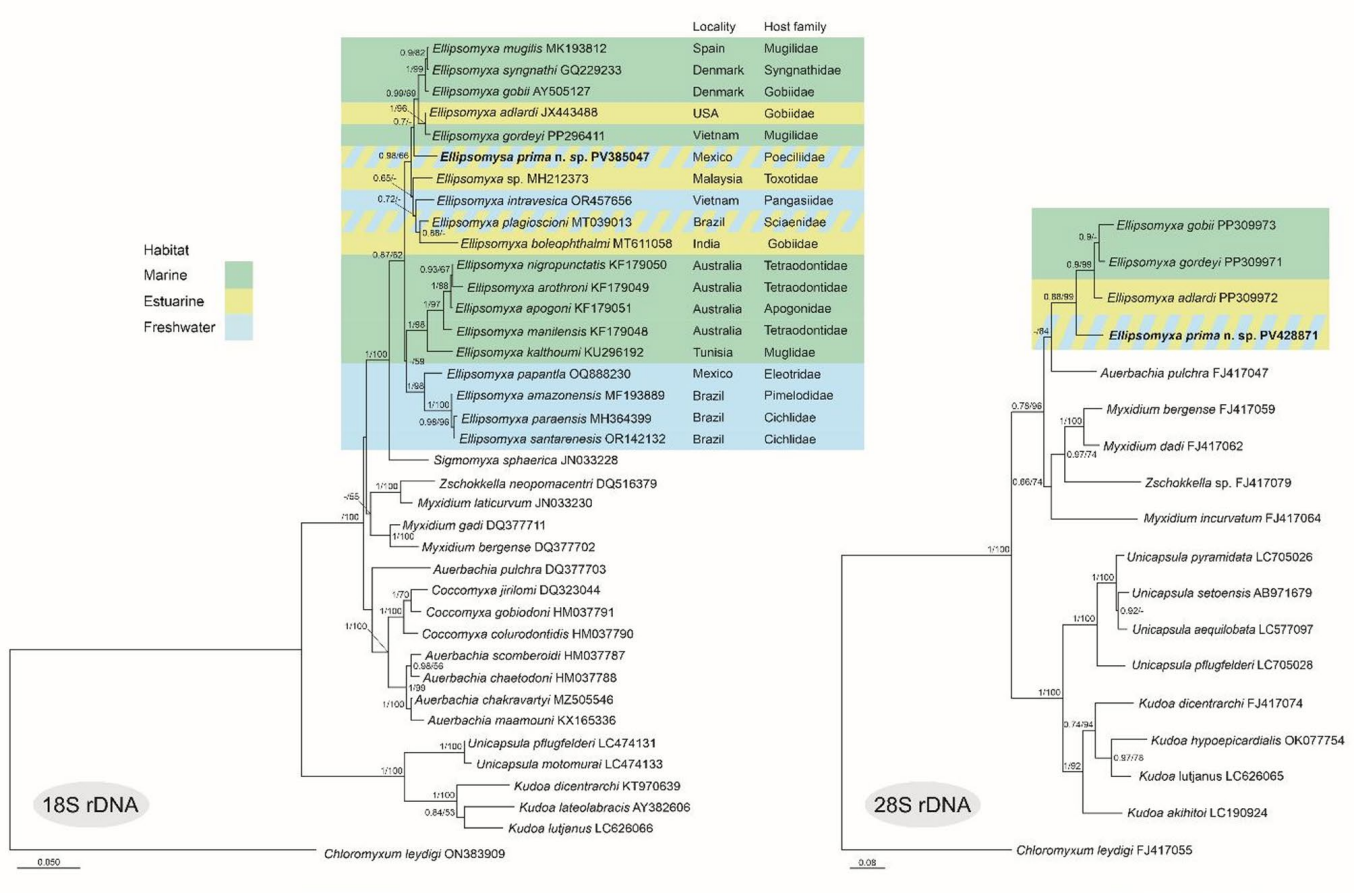
Bayesian inference phylogenetic trees from 18S and 28S rDNA for *Ellipsomyxa prima* n. sp. and its relatives. GenBank accession numbers are adjacent to the species name. Posterior probabilities (PP) and Bootstrap (BP) values greater than 50% are included at branch nodes. The scale bars represent substitutions per site.

Phylogenetic analyses of 18S rDNA and 28S rDNA sequences indicated that *E*. *prima* n. sp. is positioned within a clade that includes *Ellipsomyxa* species found in marine, estuarine, and freshwater fish hosts (Fig. 3). The 18S rDNA phylogeny showed that the *Ellipsomyxa* divided into two subclades. The smaller one comprises exclusively marine species. The bigger subclade split into two additional distinct lineages, supported only by posterior probability (PP = 1.0): one consisting of a mixture of estuarine, freshwater, and marine species, and the other comprising only freshwater species. The other subclade contains a mixture of marine, estuarine, and freshwater species (*E*. *adlardi*, *E*. *ariusi*,* E. boleophthalmi*,* E. gobii*,* E*. *gordeyi*,* Ellipsomyxa intravesica* Ksepka & Bullard, 2023, *Ellipsomyxa mugilis* Sitja-Bobadilla & Alvarez-Pellitero, 1993, *Ellipsomyxa plagioscioni* Zatti, Maia & Adriano, 2020, *Ellipsomyxa syngnathi* Køie & Karlsbakk, 2009, and *Ellipsomyxa* sp.), which includes *E*. *prima* n. sp. (PP = 0.89). However, *E*. *prima* n. sp. did not show any close relationship with any species (Fig. 3A). In the 28S rDNA phylogenetic analysis, *E*. *prima* n. sp. is a sister to a clade (PP = 0.88; BS = 99%) comprising the marine species *E. gobii* and *E. gordeyi*, and the estuarine *E. adlardi* (Fig. 3B). Due to the low number of sequences it is not possible to draw conclusions based on the phylogeny of 28S rDNA sequences.

## Discussion

*Gambusia yucatana* is an endemic freshwater fish from Southern Mexico that has been suggested as a suitable sentinel organism for ecotoxicological studies^12,13^. No parasites have been documented in this fish species to date. In the present study, we found a 100% prevalence of *Ellipsomyxa* myxospores in the gallbladders of 24 *G*. *yucatana* individuals. In most wild fish populations, the prevalence has been documented as lower, while high or full prevalence of myxozoans has been more frequently detected in fish farm settings or laboratory experiments^45,46^. However, some environmental factors, such as eutrophication, have been associated with high prevalence in wildlife fish populations^47,48^. It has been suggested that eutrophication may increase the prevalence of infected fish and the infracommunity richness of myxozoans in wildlife hosts^47,49^. It has been theorized that eutrophication may trigger an increase in invertebrate host populations, with a consequent positive effect on the development and release of infecting actinospores for fish hosts^49^. The Celestún coastal lagoon has been distinguished due to exhibiting a tendency towards eutrophication, due to often restricted water exchange with the adjacent ocean, leading to the accumulation of nutrients derived from the surrounding watershed^50^. The 100% prevalence of myxozoans associated with fish host from this lagoon has been reported for *M*. *mayarum* infecting *M*. *urophthalmus* from Celestún lagoon, and their adjacent Baldiosera freshwater spring was observed^4^. The specimens of *G*. *yucatana* examined in this study were obtained from two freshwater springs adjacent to the Celestún coastal lagoon, Baldiosera and Ya´xaa. The populations of *G*. *yucatana* from the Yucatán Peninsula region differ from those in the rest of Mexico, as many stocks in this region inhabit isolated freshwater springs or cenotes^8^. Future studies with sampling in different geographical regions of Mexico could be useful to verify the distribution of the fish and to make comparisons on the prevalence of *E*. *prima* n. sp.

The general morphology of *E*. *prima* n. sp. myxospores is consistent with the morphology of other *Ellipsomyxa* species previously described. *Ellipsomyxa gordeyi* is the closest species based on morphometric and morphological descriptions; nevertheless, the two species differ regarding the fish host, locality, and genetics. To date, more than 2,600 species of myxosporeans have been reported; however, less than 1% belong to the genus *Ellipsomyxa*, which was differentiated from *Leptotheca* Thélohan, 1895 at the beginning of the millennium^5,37^. Currently, the genus *Ellipsomyxa* is placed in the family Ceratomyxidae. However, its taxonomic position is questionable, as morphological, molecular, and phylogenetic studies have shown that *Ellipsomyxa* is closer to the genus *Myxidium* (family Myxidiidae) than the genus *Ceratomyxa*^18,44^. The 18S rDNA sequences of *E*. *prima* n. sp. showed moderate genetic sequence similarity with the available sequences reported for the nominal species (≤ 96.4%). In turn, the 28S rDNA sequence showed lower values of similarity (≤ 85.2%) with the only three available sequences of *E*. *adlardi*, *E*. *gobii*, and *E*. *gordeyi*. The genetic similarities should be handled carefully, as the available 28S rDNA sequences are shorter (~ 700 bp) than the obtained sequence of *E*. *prima* n. sp. (2216 bp), and it is unknown whether the remaining 28S rDNA sequences of the species above encompass additional variable or conservative regions over those 700 bp analysed in this study; therefore the current data may be insufficient to ensure the accuracy of which species is most genetically similar to *E*. *prima* n. sp.

Despite our molecular analyses, which include DNA sequence data of all congeneric *Ellipsomyxa*, no clear phylogenetic patterns could be observed between species, especially those collected from marine and brackish hosts. The phylogeny shows that *Ellipsomyxa* parasites have rapidly specialized, complicating the resolution of relationships within the genus. However, the present and previous results^5,6,42^ indicate that the genus *Ellipsomyxa* has a monophyletic origin. The molecular data available in the genus are still very limited, and unravelling potential evolutionary patterns requires the identification of additional species and sequences. *E*. *prima* n. sp., found in *G*. *yucatana*, is a freshwater species that can tolerate brackish water. In the 18S rDNA phylogenetic analysis, it is positioned in a separate clade with *Ellipsomyxa* species parasitizing freshwater fish (only supported by posterior probability, PP = 1.0). Similar results have been reported by Zatti et al.^42^ and Ksepka et al.^39^. In contrast, *E*. *intravesica* has been reported in the freshwater host *Pangasius macronema* Bleeker from Vietnam but phylogenetically associated with *Ellipsomyxa* species that have been reported with brackish and marine hosts^39^. Meanwhile, Zatti et al.^42^ detected the *E. plagioscioni* parasite in the gallbladder of *Plagioscion squamosissimu*s Heckel, which is mainly a freshwater fish but is commonly found also in brackish water in the Amazonian estuarine environments in South America. Phylogenetic analyses suggested that marine transgressions in the Central Region of South America probably influenced a pathway for the adaptation and rapid radiation of these cnidarian parasites in freshwater environments^42^. Similarly, the results of our phylogenetic analyses also suggest the potential for the wide distribution of the *Ellipsomyxa* species in the southern Mexican region. To elucidate this complex phylogenetic question, a larger amount of data on the *Ellipsomyxa* genus from different water environments in Mexico and other territories will need to be collected.

## Conclusion

Based on morphological characterization and molecular data of the 18S rDNA and 28S rDNA sequences, a novel myxozoan parasite, *Ellipsomyxa prima* n. sp. was described from the *Gambusia yucatana* endemic fish in Mexico. This new species is the first record of an *Ellipsomyxa* species reported within the order Cyprinodontiformes, family Poeciliid, and the first detected parasite in *G*. *yucatana*. The available information about the genus *Ellipsomyxa* is still limited. The present study indicates that *Ellipsomyxa prima* n. sp. does not form monophyletic clade with other *Ellipsomyxa* species from freshwater environments. Further studies employing molecular and morphological data on novel *Ellipsomyxa* species from different aquatic environments could clarify the evolutionary context of these parasites.

## Electronic supplementary material

Below is the link to the electronic supplementary material.

Supplementary Material 1

## Data Availability

The datasets generated and/or analysed during the current study are available in the GenBank repository, Acc. Number PV385047 (https://www.ncbi.nlm.nih.gov/nuccore/PV385047) (for data request, contact the author Graciela Colunga-Ramírez, graciela.colunga@vmri.hun-ren.hu) and PV428871 (https://www.ncbi.nlm.nih.gov/nuccore/PV428871). Type material is deposited in the parasitological collection of the Zoological Department, Hungarian Natural History Museum, Budapest, Coll. No. HNMPCC-HNHM-PAR-72085 is available through the head of the Zoological Department on reasonable request. The DNA sample is available at request (contact: Gábor Cech, cech.gabor@vmri.hun.ren.hu).
