# Supplementary material for: Description of Ellipsomyxa prima n. sp. in the gallbladder of Gambusia yucatana (Cyprinodontiformes: Poeciliidae) from freshwater springs in the Yucatán Peninsula, Mexico
Source: Sci Rep. 2025 Jul 12;15:25213. doi: 10.1038/s41598-025-10781-w (PMC12255761; doi:10.1038/s41598-025-10781-w)
Supplement: Supplementary file 1 — Supplementary Material 1 [file 41598_2025_10781_MOESM1_ESM.docx]

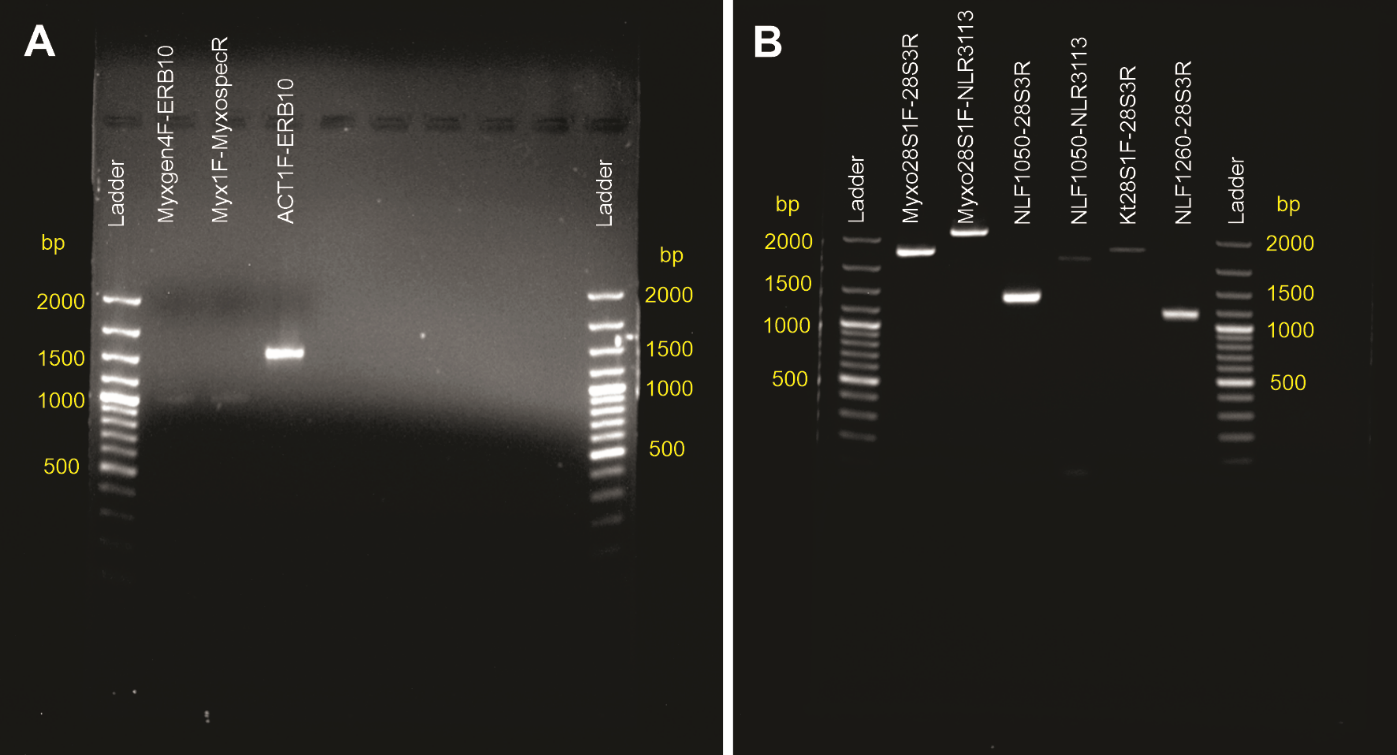
**Supplementary Fig. 1** Agarose gel electrophoresis (1%) of PCR products from *Ellipsomyxa prima* n. sp. (**A**) Amplifications of the 18S rDNA gene. (B) Amplification of the 28S rDNA gene. Base pair (bp).
